# Isoprenoids enhance the stability of fatty acid membranes at the emergence of life potentially leading to an early lipid divide

**DOI:** 10.1098/rsfs.2019.0067

**Published:** 2019-10-18

**Authors:** Sean F. Jordan, Eloise Nee, Nick Lane

**Affiliations:** Centre for Life's Origin and Evolution, Department of Genetics, Evolution and Environment, University College London, Darwin Building, Gower Street, London WC1E 6BT, UK

**Keywords:** fatty acids, protocells, origin of life, lipid divide, vesicles, isoprenoids

## Abstract

Two key problems concern cell membranes during the emergence and early evolution of life: what was their initial composition, and why did the membranes of archaea and bacteria diverge? The composition of the first cell membranes could shed light on the most likely environment for the emergence of life. The opposing stereochemistry of modern lipid glycerol-phosphate headgroups in bacteria and archaea suggests that early membranes were composed of single chain amphiphiles, perhaps both fatty acids and isoprenoids. We investigated the effect of adding isoprenoids to fatty acid membranes using a combination of UV–visible spectroscopy, confocal microscopy and transmission electron microscopy. We tested the stability of these membranes across a pH range and under different concentrations of ionic species relevant to oceanic hydrothermal environments, including Na^2+^, Cl^−^, Mg^2+^, Ca^2+^, $\mathrm{HC}O_{3}^{-}$, Fe^3+^, Fe^2+^ and S^2−^. We also tested the assembly of vesicles in the presence of Fe particles and FeS precipitates. We found that isoprenoids enhance the stability of membranes in the presence of salts but require 30-fold higher concentrations for membrane formation. Intriguingly, isoprenoids strongly inhibit the tendency of vesicles to aggregate together in the presence of either Fe particles or FeS precipitates. These striking physical differences in the stability and aggregation of protocells may have shaped the divergence of bacteria and archaea in early hydrothermal environments.

## 1. Introduction

Alkaline hydrothermal vents are among the most plausible environments for the emergence of life [1–8]. Vents are large chimney-like mineral structures that are precipitated from the fluids produced by serpentinization. Serpentinization occurs when ocean water reacts with olivine minerals in the Earth's crust forming the mineral serpentinite and producing warm (*ca* 50–100°C), alkaline (pH 9–12) fluids containing H_2_, CH_4_ and HS^−^ [1]. These warm, buoyant fluids rise to the seafloor where, upon contact with the cold seawater above, precipitate as large mineral vent structures that lack a central chimney but are instead riddled with labyrinths of interconnected micropores. In modern day alkaline hydrothermal vent systems such as Lost City and Strýtan, these precipitates are composed predominantly of carbonate or silicate minerals [9,10]. However, in the Hadaean, the combination of a ferruginous ocean and sulfur-containing fluids would have produced precipitates laced with iron–sulfur minerals such as greigite or mackinawite [1].

The ocean at that time was probably relatively acidic (*ca* pH 5–6) [11], giving rise to potentially steep pH gradients across thin inorganic barriers separating alkaline hydrothermal fluids from acidic ocean waters [1]. Proton gradients can modulate the redox potential of fluid phases, arguably facilitating CO_2_ reduction by H_2_, and thereby driving the synthesis of simple organic molecules including carboxylic acids and fatty acids [1,12]. We envisage that this process could ultimately drive protometabolism in fatty acid protocells lining the pores [13]. This picture is of course hypothetical, but prefigures autotrophic bacteria and archaea in that their carbon and energy metabolism is typically driven by dynamic ion gradients across phospholipid membranes with equivalent topology [3,14–16]. In order to harness geological proton gradients, however, simpler, more permeable membranes are required to prevent collapse of the gradient by allowing the escape or neutralization of protons that entered the protocell in the absence of active pumps [17].

Bilayer membranes formed from single chain amphiphiles (SCAs), known as lipid vesicles or protocells, likely represent the first cell membranes of life [18–22]. Phospholipids are arguably too complex to have been formed via prebiotic chemical syntheses at the time of the first protocells. SCAs, such as fatty acids, are molecules containing both hydrophilic and hydrophobic moieties, analogous to membrane phospholipids. The amphiphilic nature of these molecules favours the formation of bilayers in aqueous conditions. These microscopic (often less than 1 µm) compartments could have encapsulated the prebiotic chemical systems of a protometabolism [3,13,23]. They may also have played an active role in this protometabolism by housing simple molecular machinery, precursors of modern membrane-bound proteins [13]. Their high proton permeability is strictly necessary to take advantage of geologically sustained gradients, which is not possible in phospholipid protocells as their proton permeability is about four orders of magnitude lower [17]. However, fatty acid vesicles are able to retain other molecules much better, especially the charged molecules associated with intermediary metabolism. Enhanced organic synthesis equates to growth and division, potentially allowing the most efficient protocell lines to proliferate. These non-living entities may represent the initial steps towards the emergence of life.

Understanding the formation and stability of these membranes is therefore essential to determine whether they can perform these functions. Membranes composed of a single fatty acid will form in H_2_O, but their assembly is restricted by pH: they can only form at their apparent p*K*a, where the levels of protonated and deprotonated head groups are sufficient to allow hydrogen bonding [20]. Decanoic acid (DA) for example can only form vesicles around pH 7 [21,24,25]. Salts and divalent cations have also been shown to inhibit membrane formation. While some salt is in fact necessary [26], high concentrations can lead to aggregation of lipid molecules [27]. Various SCAs provide membranes with particular attributes. Addition of aliphatic alcohols to fatty acid membranes enhances the pH range of vesicle formation [20,24,25]. Vesicles composed of a mixture of dodecanoic acid and dodecan-1-ol can form at pH values as high as 11.6 [20]. SCAs with amine, glycerol and sulfate headgroups have been shown to increase vesicle formation in the presence of high concentrations of salts, as well as at pH values as low as 2 and as high as 10 [28]. However, the prebiotic relevance of some of these molecules remains unclear. In contrast, fatty acids and 1-alkanols have been synthesized by Fischer–Tropsch-type synthesis under hydrothermal conditions [29]. Mixtures containing six to twelve of these simple organic molecules have been shown to form vesicles under alkaline hydrothermal vent conditions including a pH range of 7–12 and high concentrations of salt and divalent cations [30]. The addition of two isoprenoid molecules enhanced the stability of these vesicles even further [30].

Isoprenoids are of interest as they form the backbone of archaeal membrane phospholipids whereas fatty acids perform this function in bacterial and eukaryotic membranes. As noted, the head group stereochemistry of these phospholipids differs as well: *sn-*glycerol-3-phosphate (G3P) and *sn-*glycerol-1-phosphate (G1P) in bacterial/eukaryotic and archaeal membrane lipids respectively. Although some ether bound lipids are found in bacteria and eukaryotes this is uncommon and they usually form only a minor proportion of the membrane [31]. To date, the majority of prebiotic vesicle research has focused on fatty acids and other aliphatic hydrocarbons while the formation of vesicles from isoprenoid molecules has been largely ignored. This is a gap in the literature as it has been suggested that the last universal common ancestor (LUCA) may have had both bacterial and archaeal membrane lipids [32]. If that were the case, then there must have been a divergence of two distinct populations after the emergence of the first living cells with mixed membranes, one group (archaea) containing predominantly isoprenoid membranes, while the other group (bacteria) having predominantly fatty acid membranes.

The prebiotic synthesis of isoprenoids remains unclear. The aqueous synthesis of carbohydrates containing the isoprene skeleton has been achieved via the formose reaction, including under mild alkaline hydrothermal conditions [33,34]. However, more work is required to determine the likelihood of these and other possible sources of isoprenoids. There has also been relatively little research on the formation of vesicles from isoprenoid SCAs [35,36]. For example, although the addition of isoprenoids to aliphatic hydrocarbon vesicles has been shown to enhance stability in an alkaline hydrothermal fluid analogue [30], the specific aspects of isoprenoid incorporation have not yet been tested.

**Figure 1.**
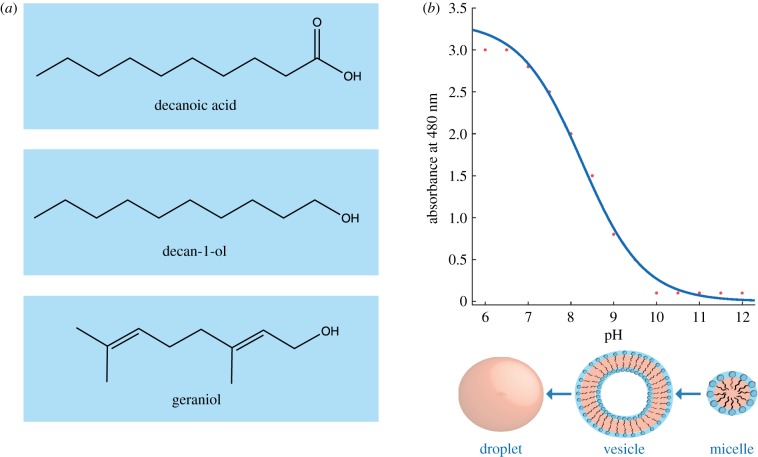
(*a*) Chemical structures of single chain amphiphiles used in this study: decanoic acid (DA), decan-1-ol (DOH) and geraniol (GOH). (*b*) Sample dataset depicting the standard sigmoid shape observed for optical density analysis of a vesicle solution. Transition between different structural states is shown below. (Online version in colour.)

Here, we aimed to assess the effects of isoprenoid addition to fatty acid vesicles. We tested the formation of vesicles in solutions containing C_10_ hydrocarbons, namely DA, decan-1-ol (DOH) and geraniol (GOH) (figure 1), across a range of pH using a combination of UV–visible spectroscopy, confocal microscopy and transmission electron microscopy (TEM). We also prepared these solutions in the presence of salts relevant to a Hadaean alkaline hydrothermal vent environment. Our results highlight the potential positive and negative aspects of isoprenoid incorporation to a prebiotic membrane compartment, shedding light on the possible composition of LUCA's cell membrane as well as a possible reason for the lipid divide.

## 2. Results

### 2.1. Formation of vesicles in H_2_O

The formation of vesicles from DA alone has been studied extensively [20,21,24,25]. DA will form vesicles around pH 7, the apparent p*K*a, when the COOH head group of the amphiphile exists as a mixture of its protonated and deprotonated forms [20]. It is known that molecular interactions can affect the p*K*a, therefore this is the apparent p*K*a of the DA system which has been measured at pH 7.1–7.3 [37]. Outside of a range of *ca* 0.2 pH units, vesicles will not form. At slightly alkaline pH DA exists in predominantly micellar form whereas at slightly acidic pH it forms hydrophobic droplets. All SCAs must be above their critical bilayer concentration (CBC) to form membranes. The CBC for DA is *ca* 40 mM [24,25,30].

**Figure 2.**
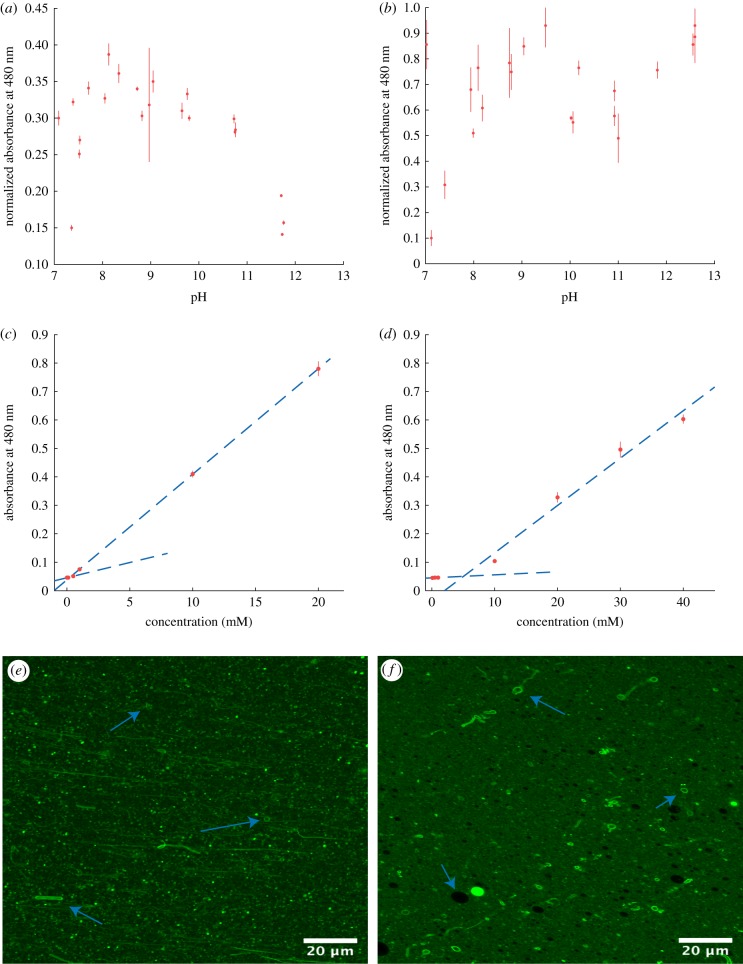
(*a*) OD data for 50 mM 1 : 1 DA/DOH solutions from pH 7 to 13. (*b*) OD data for 50 mM 1 : 1 DA/GOH solutions from pH 7 to 13. (*c*) Plot of absorbance at 480 nm versus concentration (mM) for DA/DOH. The intersection of the two trendlines indicates the CBC of 157 µM. (*d*) Plot of absorbance at 480 nm versus concentration (mM) for DA/GOH. The intersection of the two trendlines indicates the CBC of 4.9 mM. (*e*) Confocal micrograph of DA/DOH in H_2_O at pH 12.1. (*f*) Confocal micrograph of DA/GOH in H_2_O at pH 11.6. Arrows indicate individual vesicles in solution. (Online version in colour.)

The addition of 1-alkanols to fatty acid vesicles has been shown previously to increase the pH range of vesicle formation [20]. This is due to the increased hydrogen bonding provided by the presence of the OH head group. We tested the effect of the addition of DOH to DA solutions. Note that in solutions <pH 12 that were titrated with HCl, the reaction between NaOH and HCl leads to a low salt concentration (less than 150 mM). This low concentration did not appear to negatively affect vesicle formation in these mixtures. As the CBC of DA is *ca* 40 mM, a concentration of 50 mM was used for each amphiphile throughout this study to ensure enough SCA was available and results were comparable. As such, these mixtures were prepared in a 1 : 1 ratio. Vesicle formation was first tested by measuring optical density (OD) by UV–visible spectroscopy at 480 nm. Micelles scatter light minimally so minimal OD values are normally observed above the pKa of the SCAs. Vesicle formation is then indicated by increasing OD values usually forming a sigmoid shape with saturation at lower pH as droplets begin to form that scatter light maximally (figure 1). However, for DA/DOH, all solutions produced elevated OD values. Confocal microscopy confirmed that DOH addition enabled vesicle formation from pH *ca* 7 to 13, a total range of 6 pH units substantially greater than 0.2 pH units for DA alone (figure 2; electronic supplementary material, figures S1–S3).

No vesicle formation was observed below pH *ca* 6.8. In solutions above this pH confocal micrographs show numerous vesicles in solution as well as many droplets (figure 2). This is likely due to the high concentration of the solutions leading to the formation of hydrophobic structures as well as vesicles. Hydrophobic structures can also be seen in TEM images that depict dense amorphous organic material as well as individual vesicles (electronic supplementary material, figure S4). Under the vacuum of the TEM, vesicles often collapse to form ‘doughnut’ like structures whereas oil droplets tend to spread out across the grid. The CBC is calculated based on the optical density across a concentration range. Two linear trendlines are fitted to the data; one through all minimal values and the other through all increasing values. The point of intersection of these trendlines represents the CBC value. For the DA/DOH mixture the CBC was determined to be 157 µM for each individual lipid, allowing vesicle formation at much lower concentrations than a single fatty acid solution (figure 2).

A mixture of DA and GOH, a C_10_ isoprenoid alcohol, was then prepared to compare the effects of aliphatic versus isoprenoid alcohols. Similar to the previous solution, vesicles were formed from pH 7 to 13 and there were no vesicles present below pH *ca* 6.8 (figure 2; electronic supplementary material, figures S5–S7). However, compared with standard aliphatic hydrocarbon solutions, the appearance of this solution by confocal microscopy was unusual (figure 2). Many vesicles can be clearly observed, but they appear to be surrounded by a highly fluorescent and therefore hydrophobic fluid. When viewed live these vesicles are moving freely. It is possible that the excess lipid in these high concentrations preferentially forms this layer of material as opposed to the droplets that are seen in aliphatic solutions. TEM images show many individual vesicles (electronic supplementary material, figure S8). There is no evidence of the hydrophobic layer. Interestingly, although the pH range of vesicle formation was the same, the CBC of this mixed fatty acid/isoprenol solution was 4.9 mM for each lipid (figure 2). That is a *ca* 30-fold difference in the concentrations required to form vesicles.

The effect of isoprenoid addition on the encapsulation and release of the fluorescent dye pyranine by mixed fatty acid/1-alkanol vesicles was shown recently to be negligible [30]. This suggests that the overall membrane permeability of these systems is similar. Future work should aim to investigate this further by focusing on simpler vesicle models and also by developing methodology to analyse the effect of ionic strength on membrane permeability.

### 2.2. Effect of seawater on vesicle formation

Modern day seawater contains on average *ca* 600 mM NaCl, 50 mM Mg^2+^ and 10 mM Ca^2+^. As it remains unclear what the concentrations of these species may have been in the Hadaean ocean, modern values were used to test the effect these ions may have on vesicle formation. All solutions were prepared at pH *ca* 12 to reflect alkaline hydrothermal vent conditions. DA has previously been shown to be unable to form vesicles in low ionic strength solutions [27] and as such, solutions of this fatty acid alone were excluded from these experiments. It is clear that single fatty acid vesicles are not sufficiently stable to act as protocell membranes.

**Figure 3.**
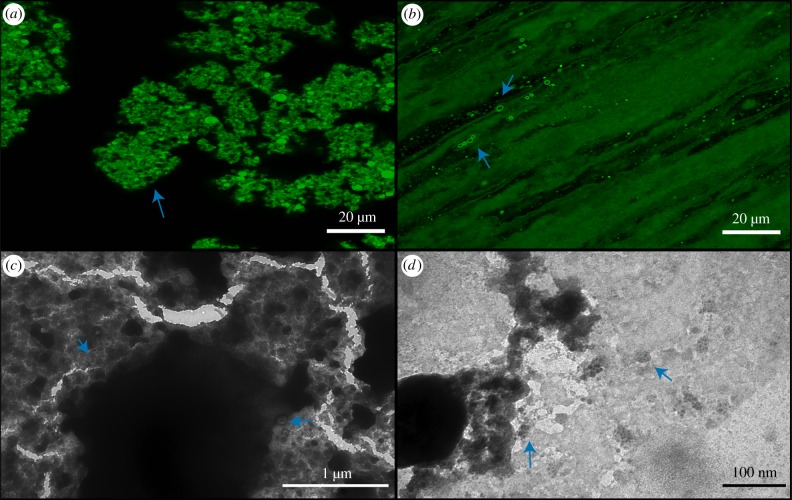
Confocal and TEM micrographs of solutions prepared in 600 mM NaCl. (*a*) Confocal micrograph of DA/DOH at pH 11.7 showing complete aggregation of vesicles, (*b*) confocal micrograph of DA/GOH at pH 11.6, (*c*) TEM micrograph of DA/DOH at pH 11.9, (*d*) TEM micrograph of DA/GOH at pH 11.8. Vesicles are indicated by blue arrows. (Online version in colour.)

The formation of individual vesicles in DA/DOH solutions was completely inhibited in 600 mM NaCl. Instead, the lipids were completely aggregated, as seen by confocal microscopy (figure 3). Many of the aggregated lipids appear to be vesicle-like; however, none of these vesicles were moving in solution, having been locked together by the presence of the high salt concentration. They have effectively been salted-out of solution. It is important to note that the fact that these aggregates still maintain some vesicle compartments may or may not be relevant to the emergence of life. In contrast, DA/GOH solutions contained numerous individual vesicles throughout (figure 3). It appears that the isoprenoid is more resistant to the salting-out effects than the 1-alkanol. The hydrophobic material observed in the H_2_O solutions was still present but appeared to be less abundant. TEM images present the vesicles as aggregated although this is due to the vacuum as opposed to a reflection of the actual solution (figure 3). Here, the hydrophobic material is visible, unlike the TEM of H_2_O samples, as indicated by the characteristic organic ‘fingerprint’ markings on the amorphous material. It is possible that although this material is sparser in the salt solutions, it is significantly denser.

**Figure 4.**
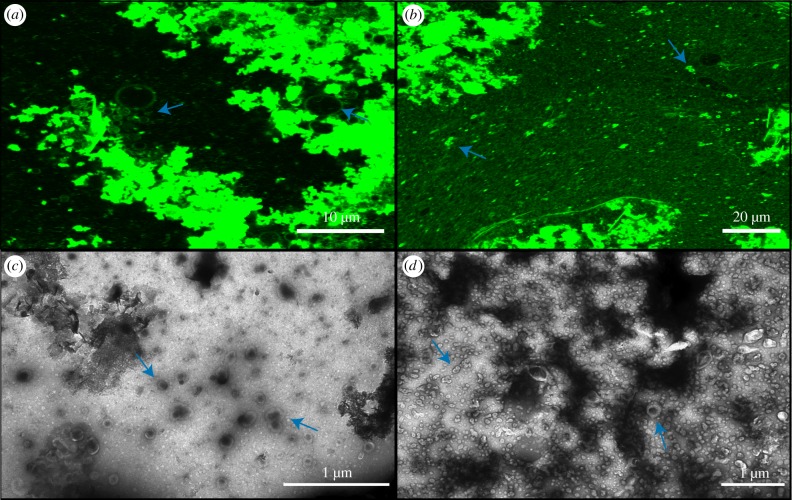
Confocal and TEM micrographs of solutions prepared in 50 mM MgCl_2_. (*a*) Confocal micrograph of DA/DOH at pH 11.2, (*b*) confocal micrograph of DA/GOH at pH 11.3, (*c*) TEM micrograph of DA/DOH at pH 11.6, (*d*) TEM micrograph of DA/GOH at pH 11.6. Vesicles are indicated by blue arrows. (Online version in colour.)

Individual vesicles are formed in solutions of 50 mM MgCl_2_ from mixtures of both DA/DOH and DA/GOH. However, both solutions also contain aggregated vesicles (figure 4). DA/DOH solutions form more aggregates than DA/GOH solutions and also contain fewer individual vesicles. DA/GOH forms some unique structures that are not observed in DA/DOH mixtures. These include filaments several micrometres in length and large rings up to 5 µm in diameter (figure 4). These structures may be composed of small vesicles or micelles, as has been shown previously [30,38–41]. The ring structures could also potentially be giant unilamellar vesicles (GUVs) that are defined as vesicles greater than 1 µm although the membranes appear thicker than average vesicles. Large ring structures up to 1 µm in diameter were observed by TEM (electronic supplementary material, figure S9). These were not composed of vesicles but had very thin borders with clear interiors, some even containing small vesicles. It is possible that these rings are composed of micelles, below the resolution of the microscope, although this is as yet unclear. There were also individual vesicles present. In TEM images of DA/DOH solutions, many individual vesicles were observed as were aggregates reflecting the results from confocal microscopy (figure 4). Some of the vesicles had quite thick membranes, up to 50 nm, presumably multi-lamellar.

**Figure 5.**
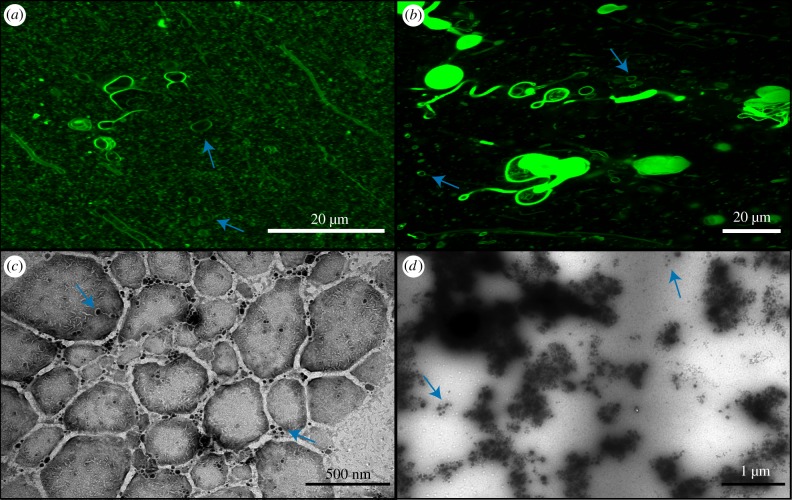
Confocal and TEM micrographs of solutions prepared in 10 mM CaCl_2_. (*a*) Confocal micrograph of DA/DOH at pH 11.6, (*b*) confocal micrograph of DA/GOH at pH 11.6, (*c*) TEM micrograph of DA/DOH at pH 11.9, (*d*) TEM micrograph of DA/GOH at pH 11.8. Vesicles are indicated by blue arrows. (Online version in colour.)

The presence of 10 mM CaCl_2_ affected vesicle formation in both DA/DOH and DA/GOH solutions. Many individual vesicles formed from DA/DOH, as seen by confocal microscopy, but there was also a large amount of fluorescent, ‘background’ material that could potentially have been vesicles below the resolution of the microscope (figure 5). Alternatively, this may be hydrophobic material similar to that observed in isoprenoid containing solutions. Some bright filament and ring-like structures were also present. TEM analysis showed that these samples did contain a large amount of hydrophobic material without any apparent structure (figure 5). This mat of material contained net-like structures composed of a mixture of vesicles and organic substance. There were also individual vesicles observed throughout. DA/GOH solutions contained numerous individual vesicles as well as large oily droplets and many filament and ring-like structures (figure 5). TEM images contained individual vesicles as well as large dark areas indicative of heavy negative staining, possibly on the large hydrophobic structures observed by confocal microscopy (figure 5).

### 2.3. Vesicle formation in alkaline hydrothermal vents

For protocell membranes to be stable in alkaline hydrothermal vent environments they would have been required to deal with the presence of additional ionic species to those present in the open ocean. The concentrations of these ions are difficult to constrain so we opted for values at the higher end of current estimates [42,43]. We tested vesicle formation in DA/DOH and DA/GOH solutions in the presence of 50 mM $\mathrm{HC}O_{3}^{-},$ 1 mM Fe^3+^, 1 mM Fe^2+^, and 1 mM S^2−^. We also tested these solutions in the presence of FeS precipitates and Fe particles to investigate the effects of solid materials potentially present in the vents on vesicle formation. $\mathrm{HC}O_{3}^{-}$ and Fe^3+^ solutions were prepared as normal while all other solutions were prepared in an anaerobic chamber with O_2_ levels maintained at 0–5 ppm. This was to prevent the oxidation of these O_2_ sensitive species. DA/DOH and DA/GOH solutions were also prepared in H_2_O within the chamber to ensure that vesicle formation was not affected by the anoxic conditions. It was not possible to maintain anaerobic conditions during confocal microscopy and TEM; however solutions were kept sealed and only exposed to air immediately prior to imaging.

**Figure 6.**
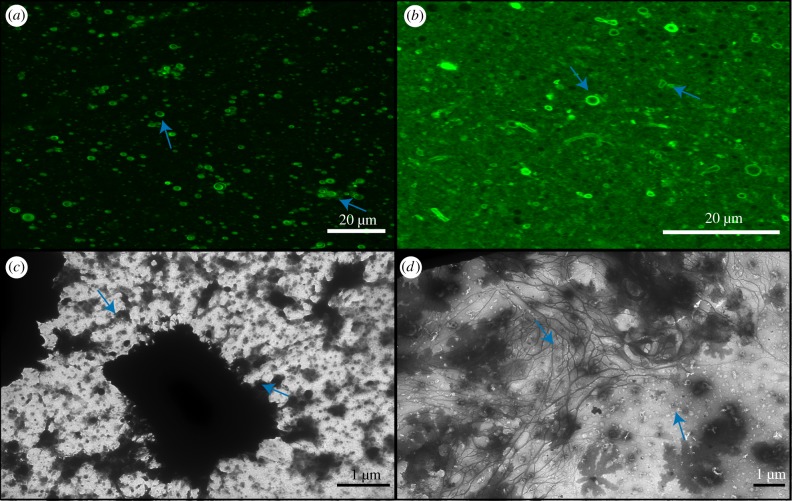
Confocal and TEM micrographs of solutions prepared in 1 mM FeCl_3_. (*a*) Confocal micrograph of DA/DOH at pH 13.0, (*b*) confocal micrograph of DA/GOH at pH 12.9, (*c*) TEM micrograph of DA/DOH at pH 12.0, (*d*) TEM micrograph of DA/GOH at pH 12.0. Vesicles are indicated by blue arrows. (Online version in colour.)

The presence of Na_2_HCO_3_ seems to have no effect on vesicle formation. Both DA/DOH and DA/GOH mixtures contain vesicles (electronic supplementary material, figures S10 and S11). DA/DOH solutions contained some GUVs up to 10 µm in diameter. TEM images of these solutions reflected the confocal microscopy (electronic supplementary material, figures S12 and S13). Both DA/DOH and DA/GOH form numerous vesicles in the presence of FeCl_3_ (figure 6). DA/GOH solutions contain the characteristic layer of hydrophobic material as well as vesicles. Some larger multilamellar vesicles are present in the DA/DOH solutions. Both solutions present interesting structures in TEM images (figure 6). DA/DOH images contain individual vesicles as well as large rectangular structures, likely crystals of some sort. Membranous material seems to be attracted to these crystals as it can be seen tethered to the edges of the rectangle. DA/GOH solutions contain material in unique dendritic patterns. It is possible that this is excess lipid material although it is difficult to interpret. Individual vesicles are dispersed throughout the grid as well.

Anoxic solutions prepared in H_2_O appeared the same as those prepared under normal laboratory conditions (electronic supplementary material, figures S14 and S15). These had vesicles in solution and DA/GOH solutions still contained the characteristic hydrophobic layer. This is also true for those solutions containing FeCl_2_ and Na_2_S (electronic supplementary material, figures S16–S19). The only apparent variation was the presence of DA/DOH GUVs in the presence of FeCl_2_. Interestingly, DA/GOH in FeCl_2_ had begun to take on an orange colour prior to exposure to O_2_, indicating oxidation of the Fe^2+^ to Fe^3+^ although the same was not observed for DA/DOH. This could be due to the reduction of the double bond in the isoprenoid molecule.

### 2.4. Vesicles in the presence of particulates

**Figure 7.**
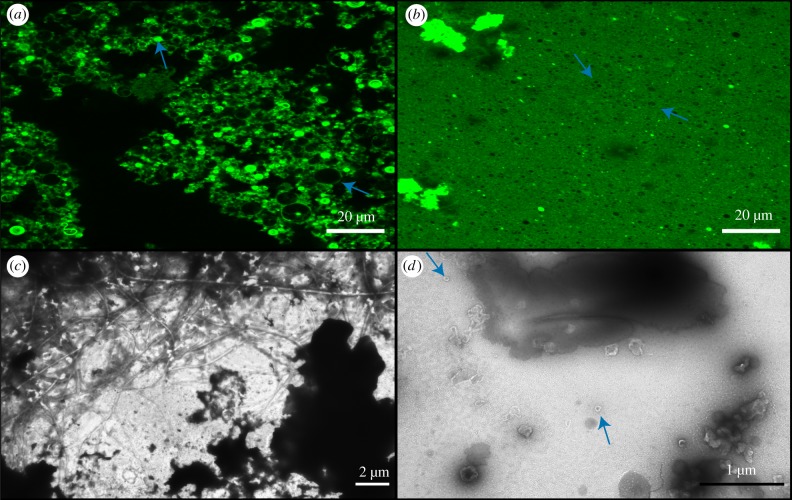
Confocal and TEM micrographs of anoxic solutions prepared in 12.5 mM FeS_2_. (*a*) Confocal micrograph of DA/DOH at pH 12.0, (*b*) confocal micrograph of DA/GOH at pH 12.0, (*c*) TEM micrograph of DA/DOH at pH 12.1, (*d*) TEM micrograph of DA/GOH at pH 12.2. Vesicles are indicated by blue arrows. (Online version in colour.)

DA/DOH and DA/GOH solutions were prepared in a mixture of 500 µM FeCl_2_ and 500 µM Na_2_S. No visible FeS precipitate was formed and both solutions appeared as standard with plentiful vesicles (electronic supplementary material, figures S20 and S21). The DA/GOH solutions again gained an orange hue prior to O_2_ exposure while this did not occur in the DA/DOH solution. The presence of the isoprenoid appears to lead to increased oxidation. In order to ensure the presence of precipitate, 12.5 mM of FeCl_2_ and Na_2_S were used to prepare vesicle solutions. These solutions were black in colour due to the large amounts of precipitate. The presence of this precipitate caused complete aggregation of DA/DOH vesicles (figure 7). No individual vesicles were observed in solution. Some of the vesicles within the aggregates were GUVs as large as 10 µm in diameter. It was not possible to distinguish individual vesicles from the dense amorphous organic material observed by TEM (figure 7). Large filamentous structures were also present in TEM images. DA/GOH vesicles appeared to be largely unaffected by the FeS precipitate (figure 7). Dark shadows could be seen in the confocal micrographs indicative of precipitate particles. Some, but not all, of these particles had small clusters of aggregated vesicles connected to them (figure 7). This was the only variation from a standard DA/GOH solution. Many individual vesicles could be seen around FeS particles in TEM images (figure 7). As in the confocal micrographs, some aggregation near the particles was also observed.

**Figure 8.**
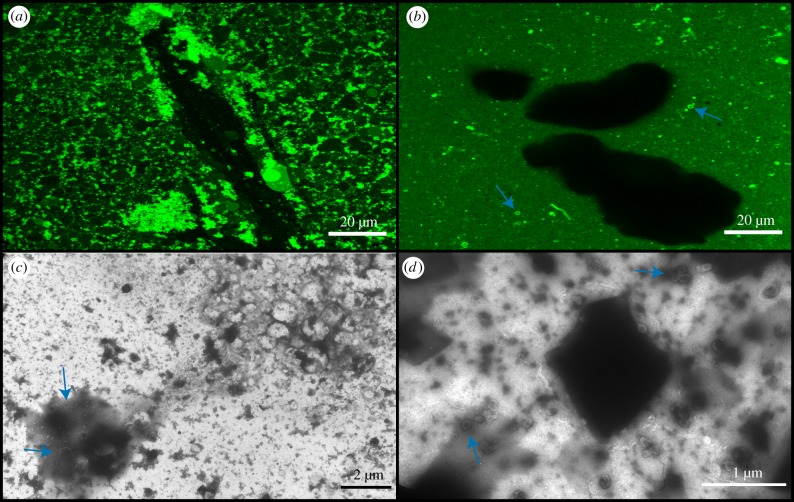
Confocal and TEM micrographs of anoxic solutions prepared in H_2_O followed by addition of Fe powder (less than 212 µm mesh). (*a*) Confocal micrograph of DA/DOH at pH 12.1, (*b*) confocal micrograph of DA/GOH at pH 12.1, (*c*) TEM micrograph of DA/DOH at pH 12.2, (*d*) TEM micrograph of DA/GOH at pH 12.1. Vesicles are indicated by blue arrows. (Online version in colour.)

Addition of Fe powder (less than 212 µm particle size) to solutions of DA/DOH in H_2_O also caused complete aggregation of vesicles (figure 8). No individual vesicles were observed by confocal microscopy. Large particles of Fe could be observed as large dark patches between the vesicle aggregates. There was also a clear association between these particles and vesicles. Dense clusters of aggregated vesicles could be observed attached to the exterior of these Fe particles. Even in areas devoid of particles, all vesicles existed as aggregates. Interestingly, some individual vesicles were present in TEM images (figure 8). Aggregated organic structures were also observed containing circular voids. These may represent some of the aggregates observed by confocal microscopy. They did not appear to be composed of vesicles although some vesicles were observed in the vicinity of the material. In contrast, DA/GOH solutions appeared largely unaltered by the Fe particles (figure 8). Individual vesicles were observed in solution as well as the characteristic hydrophobic material. Fe particles could be seen floating throughout the solutions although there was minimal interaction with vesicles. Fe particles could be seen in TEM images with individual vesicles nearby (figure 8). No aggregation was observed. In general, DA/GOH vesicles did not appear to have an attraction to the Fe particles.

## 3. Discussion

Deep sea alkaline hydrothermal vents are a promising prospect for the environment in which life first emerged. Proton gradients, sustained by the continuous flow of alkaline fluids separated from the acidic ocean by inorganic barriers, supplied a geochemical energy source prefiguring the ion gradients that drive carbon and energy metabolism in modern cells [3]. The microporous labyrinths within vents provide a concentration mechanism for organic molecules via thermophoresis while also giving shelter to protocells, allowing them to grow and divide without being washed into an open ocean [44–46]. The vent interior itself would have experienced a range of pH values, from highly alkaline at its source on the seafloor to relatively acidic nearest the vent–ocean interface. This would have resulted in multiple microenvironments that may have acted as a selection pressure on protocells and eventually the first cells. However, these conditions require bilayer membranes that are stable in these respective microenvironments.

We have shown that the addition of a C_10_ isoprenoid alcohol to a C_10_ fatty acid will form vesicles in aqueous solutions across a pH range of 7–13 (figure 2). This is the same as the range observed for DA/DOH vesicles, showing that there is no disadvantage to isoprenoid incorporation based on pH (figure 2). However, while DA/DOH vesicles will form at SCA concentrations as low as 157 µM, DA/GOH solutions require an SCA concentration of at least 4.9 mM to form vesicles, *ca* 30-fold greater (figure 2). In alkaline saline solutions containing 600 mM NaCl, GOH prevents the aggregation of vesicles and results in a solution of free-floating vesicles (figure 3). This level of salinity causes complete vesicle aggregation in DA/DOH solutions. In general, the presence of Mg^2+^, Ca^2+^, Fe^3+^, $\mathrm{HC}O_{3}^{-},$ Fe^2+^ and S^2−^ had little or no effect on the formation of vesicles in either of these SCA mixtures. However, FeS and Fe particles, two species that would likely have existed in Hadaean alkaline hydrothermal vents, led to the complete aggregation of DA/DOH vesicles while DA/GOH vesicles remained largely unaffected (figures 7 and 8). The DA/DOH aggregates are still composed of individual vesicles with bilayer membranes and aqueous interiors. Whether or not these structures could perform the functions required for protocells at the emergence of life warrants further investigation. The quantity of vesicles formed throughout these experiments varied greatly, but the fact that vesicles can form at all in a range of extreme environments is important in terms of the potential for protocells to develop.

The presence of various ionic species, particularly divalent cations, is claimed to have a detrimental impact on vesicle formation [27]. This claim segues into the generalization that the intolerance of SCAs to high ionic strength requires a freshwater environment for the origin of life [47]. This conclusion is based on evidence from mixtures containing one, two or three SCAs, predominantly aliphatic hydrocarbons. Our DA/DOH results support the idea that these simple mixtures do not form individual vesicles in any oceanic environment. However, the likelihood of such a clean hydrocarbon solution existing on a prebiotic Earth is low. Sources of organic molecules on the early Earth have been widely investigated and they were likely numerous [48]. Of particular relevance here, hydrothermal Fischer–Tropsch-type synthesis produces fatty acids and 1-alkanols with chain lengths from C_6_ to C_34_ [29]. As such, the prebiotic vent environment likely contained a high diversity of SCAs.

More complex mixtures of six to twelve aliphatic SCAs (C_10_–C_15_) have been shown to form vesicles under oceanic alkaline hydrothermal vent conditions [30]. As in the results presented here, incorporation of GOH to those mixtures further improved their stability. Aliphatic hydrocarbons with novel headgroups such as amines and sulfates have also been shown to improve stability at different pH values, predominantly ≤ pH 7, and in the presence of some salts [28]. It is clear that increased variety in bilayer membrane composition allows for vesicle formation in virtually any environment, whether marine or terrestrial. However, more work is needed to determine the possible pathways for prebiotic synthesis of varying SCAs including isoprenoids. It is unlikely that mixtures of three or less SCAs are representative of conditions on the early Earth. As such, the exclusion of any environment as a potential location for the emergence of life based on results from laboratory experiments involving vesicles composed of these simple mixtures is unreasonable.

The interaction between vesicles of varied composition and diverse mineral surfaces is also of great relevance to the origin of life. Previous work has found that multiple surface types actually promote vesicle formation [49]. We have shown here that vesicles containing isoprenoids are less likely to adsorb onto surfaces, whereas those containing aliphatic hydrocarbons alone tend to form aggregates of vesicles (figures 7 and 8). In an alkaline hydrothermal environment this aggregation may in fact be beneficial. If protocells adhered to the FeS walls of the vent they may have been in a more advantageous position for harnessing the geological proton gradient. However, if they remain aggregated even after significant growth, there is no avenue for migration and colonization of new environments. Perhaps novel SCAs synthesized within aliphatic hydrocarbon vesicles fuelled by a proton gradient would have been incorporated into the membranes allowing for their eventual detachment from the overall aggregate. There is still a paucity of research involving the interaction between vesicles, minerals and the structural assemblages they form. Given the likelihood of these interactions in all prebiotic environments, future work will need to focus on investigations of this sort.

Because isoprenoids enhance the stability of prebiotic membranes and might have formed part of the earliest living cell membranes including LUCA, it is unclear why membrane composition diverged in archaea and bacteria. Earlier work suggested that isoprenoid ether bound phospholipids enhance stability in extreme environments [50]. The idea assumed that archaea are predominantly extremophiles. However, bacteria also proliferate in these harsh environments and archaea are now known to be widespread in mesophilic niches [31,51]. In extant cells the advantages and disadvantages of each phospholipid class seem to be small. It is possible that in ancient membranes, before phosphate headgroups, the chemical differences between isoprenoid and aliphatic SCAs were sufficient to drive divergence based on environmental pressures. Here we report striking differences between these two SCA types. The differences could reflect the presence of both double bonds and branching in isoprenoids. Further work incorporating unsaturated 1-alkanols (e.g. dec-4-en-1-ol) and branched 1-alkanols (e.g. 2-methyldecan-1-ol) into these vesicle systems may allow us to determine the source of this unique chemical behaviour by a process of elimination. The length of the membrane forming SCAs may also have an impact on vesicle formation and stability. Substituting farnesol, a C_12_ isoprenoid alcohol, for GOH in these simple models could shed light on the possible effects of SCA length.

The enhanced stability of isoprenoids under hydrothermal conditions could have favoured the proliferation of protocells in which they were incorporated. Conversely, the *ca* 30-fold higher concentration of isoprenoid SCAs needed for vesicle formation, shown here through CBC analyses (figure 2), may have driven membrane divergence depending on the environment. The enzymes required for the synthesis of archaeal (G1P) and bacterial (G3P) phospholipid headgroups are phylogenetically unique. This suggests that they arose independently, which is consistent with the idea of an early lipid divide in SCA membranes [17,52]. The advent of phospholipids would then have allowed bacteria to recolonize more extreme environments relatively early. At this stage it is unlikely that there would have been a benefit to evolving new mixed membranes of both phospholipid types. Lateral gene transfer would account for the presence of both biosynthetic pathways in some modern organisms [53,54].

Previous studies have shown that liposomes formed from a mixture of archaeal and bacterial phospholipids are chemically stable in the laboratory [55,56]. The potential for engineering bacterial cells to incorporate archaeal lipids into their cell membranes has also been investigated and has proved successful [57–60]. Recently, it has been shown that *Escherichia coli* bacteria engineered to biosynthesize archaeal isoprenoid ether lipids incorporated up to 30% of these in their cell membranes [59]. These mixed membrane bacteria were stable in the laboratory for up to 6 days. Throughout this period the proportion of isoprenoid ether lipids in the membranes increased, as did cell robustness. These findings sharpen the paradox. If there is no obvious penalty for mixing lipids, then why did the lipid divide occur? One possibility is that membrane proteins rather than lipids drove the divergence [61]. While appealing, this idea is undermined by the propensity of membrane proteins to evolve rapidly [62], which could compensate for subtle differences in lipid chemistry. The present study suggests that the primary lipid divide occurred in early protocells, reflecting striking physical differences in SCA chemistry, rather than more subtle differences in phospholipid membranes. If so, any instability produced by membrane proteins would explain why phospholipid membranes never re-mixed later on, rather than the primary divide itself. The number of possible membrane compositions of both aliphatic and isoprenoid SCAs is vast, while the advantages or disadvantages of these SCA combinations have barely been studied. The startling differences between aliphatic and isoprenoid membranes reported here merely hint at their potential contribution to the lipid divide.

## 4. Conclusion

Isoprenoids enhance the stability of aliphatic SCA membranes under alkaline hydrothermal conditions by preventing aggregation in the presence of various ionic species and mineral particles. But this stability comes at a cost as much higher concentrations of isoprenoids than aliphatic hydrocarbons are required for membrane formation. The membrane of LUCA was arguably composed of both aliphatic and isoprenoid SCAs. Such mixed membranes enhance stability under alkaline hydrothermal conditions. The advantages and disadvantages of incorporating isoprenoids into cell membranes in different microenvironments may have driven membrane divergence, with the later biosynthesis of phospholipids giving rise to the unique G1P and G3P headgroups of archaea and bacteria respectively. If so, the properties conferred by membrane isoprenoids place the lipid divide as early as the origin of life.

## 5. Material and methods

### 5.1. Materials

All reagents used in this study were of analytical grade (greater than or equal to 97%) and were purchased from either Sigma Aldrich (Merck, UK) or Acros Organics (UK).

### 5.2. Preparation of vesicle solutions

Vesicle solutions were prepared following a modified version of the method described by Monnard & Deamer [24]. All solutions were prepared in glass vials in a dry heating block at 70°C. This temperature reflects the temperature observed in alkaline hydrothermal vents (50–100°C). Lipids were heated and added to solutions in liquid form. An aliquot of DA was added to deionized (DI) H_2_O and vortexed. 1 M NaOH was added until the solution turned clear, indicating complete deprotonation of the acid. The alcohol (DOH/GOH) was then added and the solution vortexed. The pH was adjusted with 1 M HCl or 1 M NaOH to achieve the desired final value. The solution was then brought to the appropriate final volume with DI H_2_O. Solutions were analysed immediately after preparation.

For the preparation of vesicles in salt solutions, H_2_O was replaced with the desired salt concentration and acid and base solutions were replaced with the desired salt dissolved in 1 M HCl and 1 M NaOH respectively to ensure constant salt concentrations throughout. Anoxic vesicle solutions (H_2_O, FeCl_2_, Na_2_S, FeS, Fe particles) were prepared following the above method in an anaerobic hood containing a 5% H_2_ in N_2_ atmosphere. O_2_ was removed by reaction with H_2_ to form H_2_O vapour on a Pd catalyst. O_2_ levels were monitored and maintained at 0 ppm throughout these procedures. FeS solutions were prepared using a 1 : 1 ratio of FeCl_2_ and Na_2_S. Fe particles were added to the relevant solutions after vesicle formation in H_2_O. All anoxic solutions were sealed with lids and wrapped in parafilm. The seal was broken immediately prior to imaging.

For CBC solutions, a stock was prepared following the same procedure and adjusted to pH *ca* 8. A range of concentrations were then prepared by serial dilution of this stock. These were analysed immediately by UV–visible spectroscopy after preparation.

### 5.3. UV–visible spectroscopy

Optical density measurements for pH range and CBC determination were performed in Falcon Costar black 96-well plates using an Infinite M200 Pro spectrophotometer (Tecan). Plates were maintained at 70°C and measurement was performed immediately at 30°C (determined previously to be suitable for vesicle analysis [30]). Plates were shaken for 5 s and scanned 25 times at 480 nm. Analysis was performed on triplicate 50 µl aliquots of each solution.

### 5.4. Confocal microscopy

Confocal microscopy was performed with a Zeiss LSM-T-PMT 880 coupled to an Airyscan detector. Membranes were imaged using a hydrophobic dye, Rhodamine 6G. Briefly, 0.5 µl of 100 µM dye was added to a heated (70°C) microscope slide. Vesicle solution was vortexed and a 5 µl aliquot was added to the slide and mixed with the dye. The mixture was covered with a #1.5 16 mm diameter coverslip and placed on the microscope stage. The dye was excited with an Ar laser operating at 514 nm and observed through a 63× oil objective with a 488 nm filter. Images were captured using Zeiss Zen microscopy software and final processing was performed using the FIJI software package.

### 5.5. Negative staining transmission electron microscopy

Negative staining TEM (NS-TEM) was performed with a JEOL 1010 TEM (JEOL, Japan). Samples were added to a Cu 100 mesh grid and allowed to sit for 30 s. Excess sample was then blotted away with filter paper and an aliquot of aqueous uranyl acetate (1.5%) was added to the grid. This was allowed to stand for 30 s and then blotted. Grids were analysed immediately under vacuum. Image processing was performed using the FIJI software package.

## Supplementary Material

Supplementary Information

## Data accessibility

This article has no additional data.
