# Supplementary material for: Isoprenoids enhance the stability of fatty acid membranes at the emergence of life potentially leading to an early lipid divide
Source: Interface Focus. 2019 Oct 18;9(6):20190067. doi: 10.1098/rsfs.2019.0067 (PMC6802135; doi:10.1098/rsfs.2019.0067)
Supplement: Supplementary Information [file rsfs20190067supp1.docx]

**Supporting Information**

Isoprenoids enhance the stability of fatty acid membranes at the emergence of life potentially leading to an early lipid divide

Sean F. Jordan^1*^, Eloise Nee^1^, Nick Lane^1^

^1^ *Centre for Life’s Origin and Evolution, Department of Genetics, Evolution and Environment, Darwin Building, Gower Street, University College London, London WC1E 6BT, UK*

*Corresponding author: *E-mail address:* [sean.jordan@ucl.ac.uk](mailto:sean.jordan@ucl.ac.uk) (Sean F. Jordan)

**Results**

Table 1. Critical bilayer concentration (CBC) values for DADOH (n = 3)

| DADOH CBC Values | | |
| --- | --- | --- |
| Concentration (mM) | Mean absorbance | Standard Deviation |
|  |  |  |
| 20 | 0.780 | 0.026 |
| 10 | 0.410 | 0.012 |
| 1 | 0.075 | 0.001 |
| 0.5 | 0.051 | 0.001 |
| 0.1 | 0.046 | 0.000 |
| 0 | 0.046 | 0.000 |

Table 2. Critical bilayer concentration (CBC) values for DADOH (n = 3)

| DAGOH | | |
| --- | --- | --- |
| Concentration (mM) | Mean absorbance | Standard Deviation |
|  |  |  |
| 40 | 0.603 | 0.016 |
| 30 | 0.496 | 0.028 |
| 20 | 0.328 | 0.018 |
| 10 | 0.104 | 0.002 |
| 1 | 0.046 | 0.001 |
| 0.5 | 0.046 | 0.001 |
| 0.1 | 0.045 | 0.001 |
| 0 | 0.045 | 0.000 |


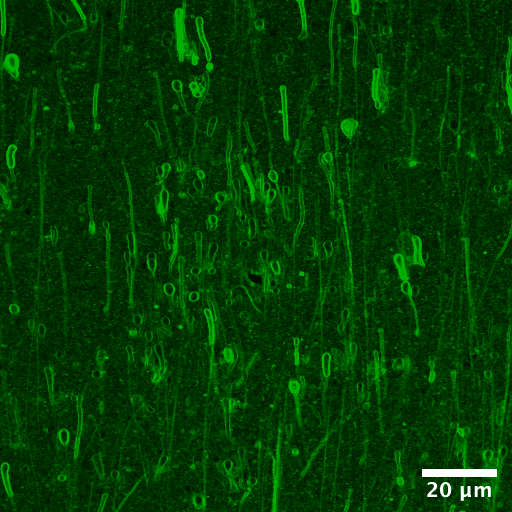
Figure 1. Confocal micrograph of DADOH in H_2_O at pH 7.8.
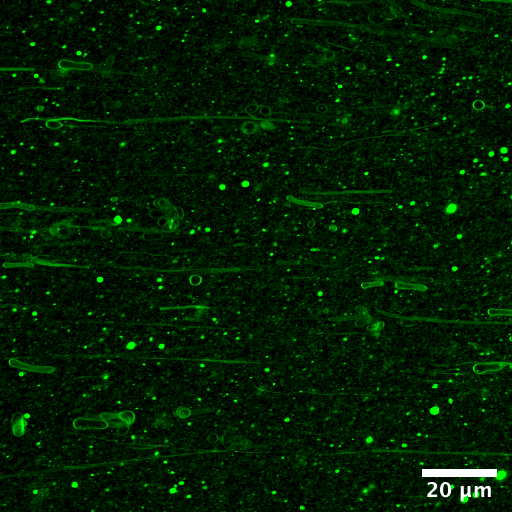
Figure 2. Confocal micrograph of DA/DOH in H_2_O at pH 8.6.
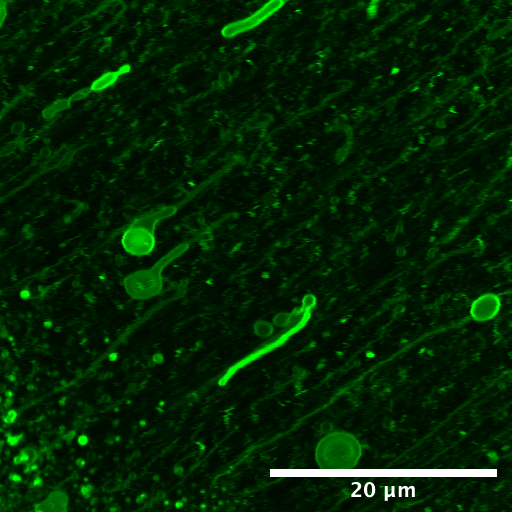
Figure 3. Confocal micrograph of DA/DOH in H_2_O at pH 12.1.
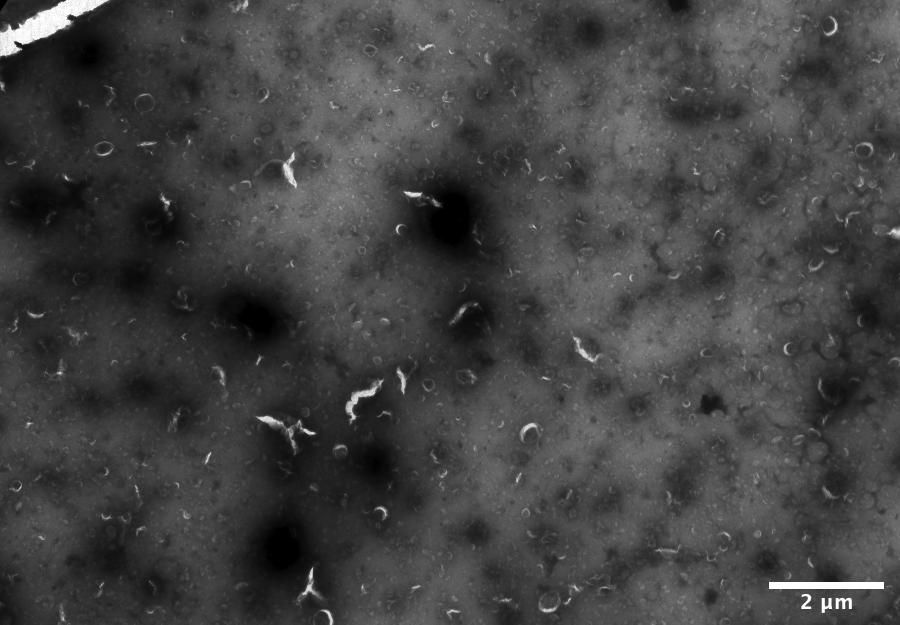
Figure 4. TEM micrograph of DA/DOH in H_2_O at pH 12.1.
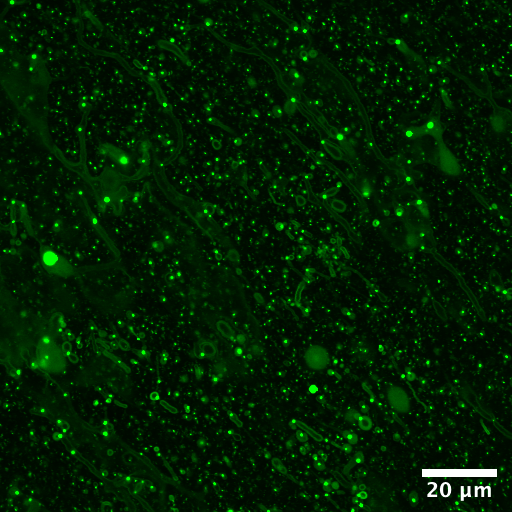
Figure 5. Confocal micrograph of DA/GOH in H_2_O at pH 7.4.
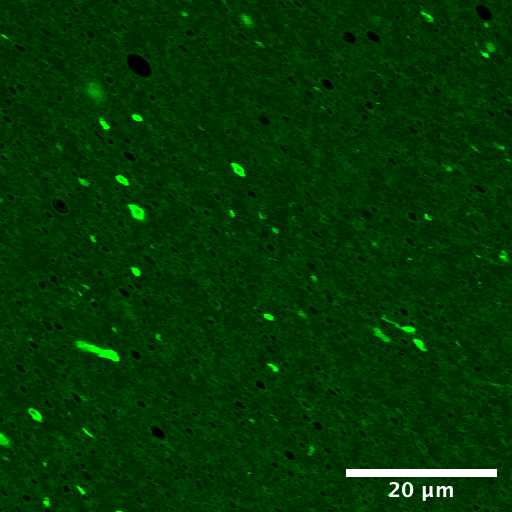
Figure 6. Confocal micrograph of DA/GOH in H_2_O at pH 8.8.
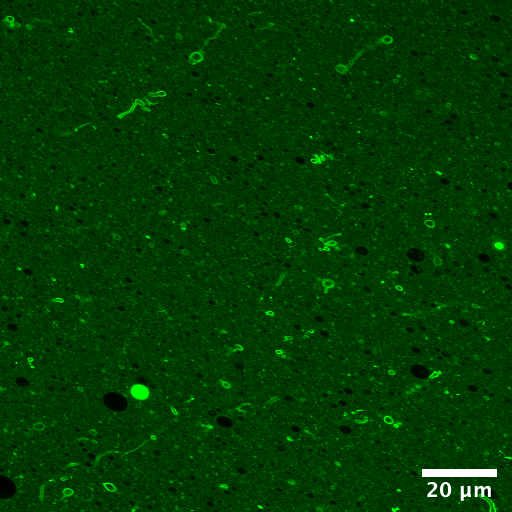


Figure 7. Confocal micrograph of DA/GOH in H_2_O at pH 11.6.


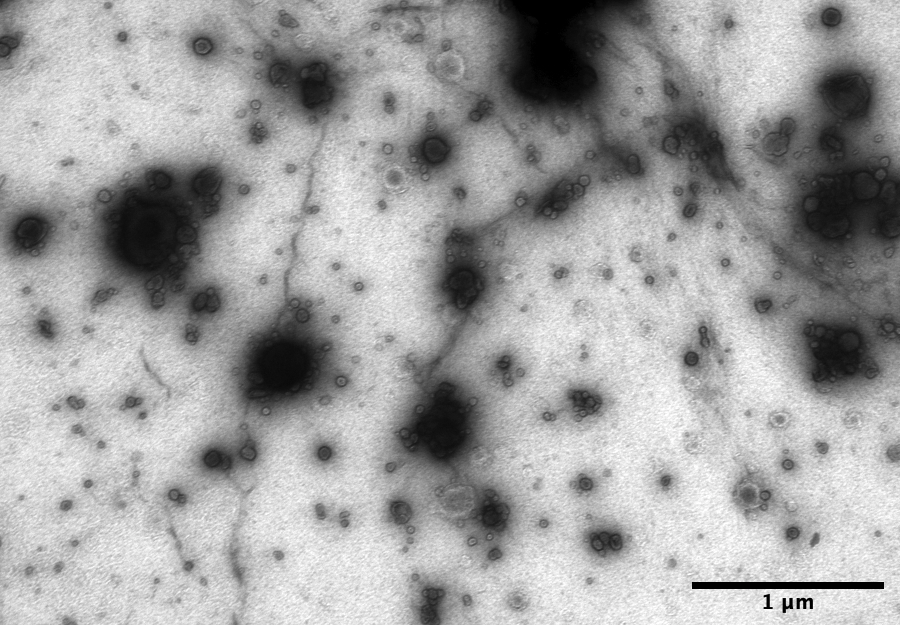
Figure 8. TEM micrograph of DA/GOH in H_2_O at pH 12.1.


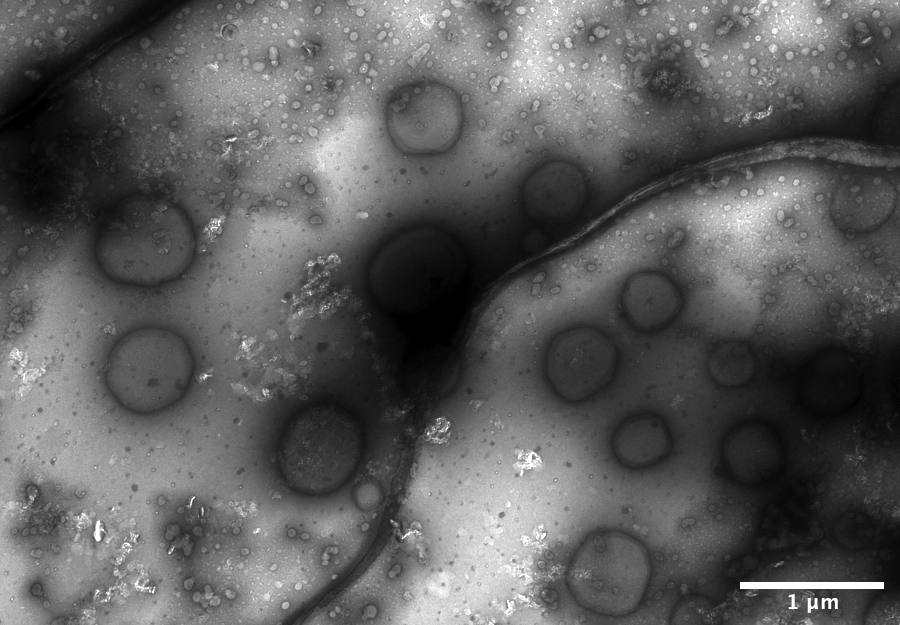
 Figure 9. TEM micrograph of DA/GOH in 50 mM MgCl_2_ at pH 11.6.
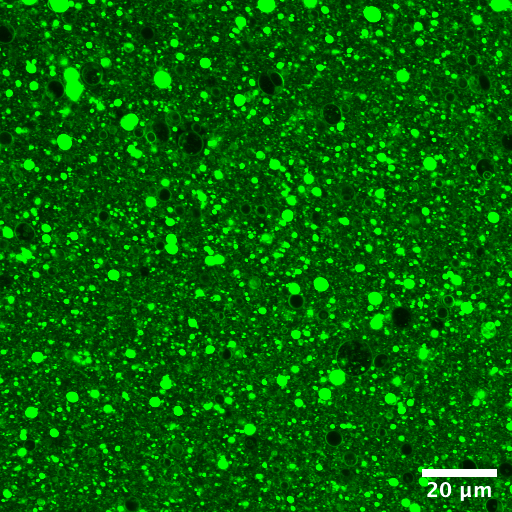
Figure 10. Confocal micrograph of DA/DOH in 50 mM NaHCO_3_ at pH 12.9.
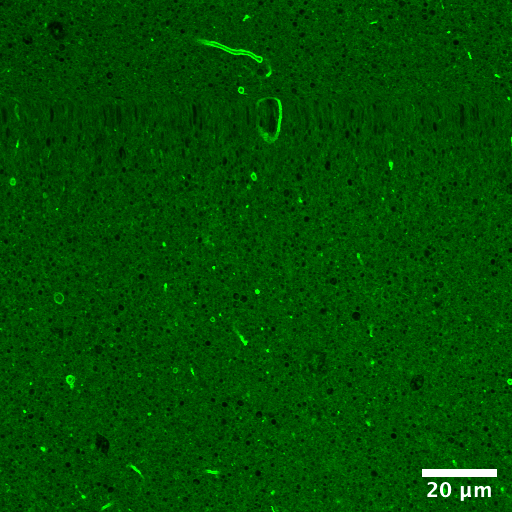
Figure 11. Confocal micrograph of DA/GOH in 50 mM NaHCO_3_ at pH 12.9.
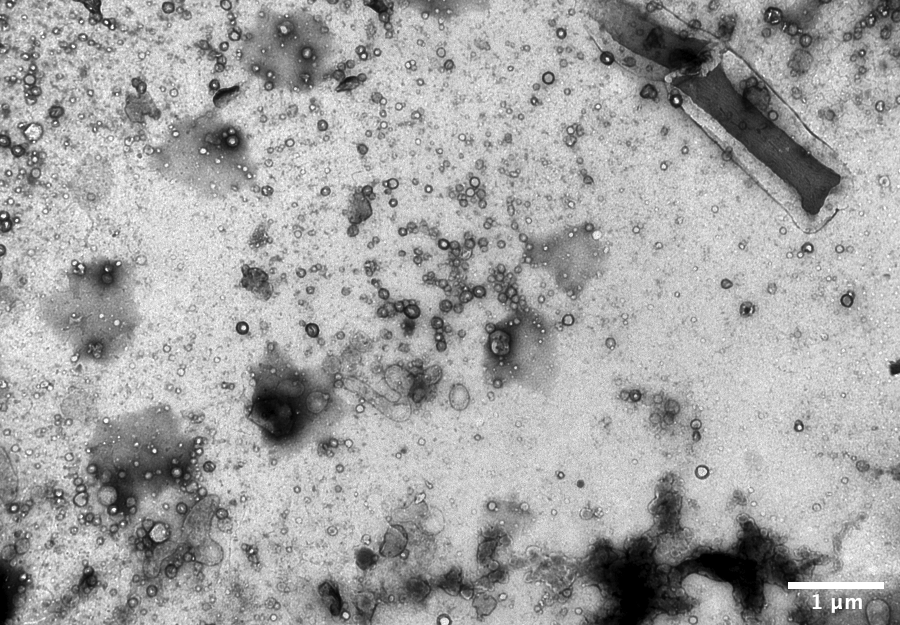


Figure 12. TEM micrograph of DA/DOH in 50 mM NaHCO_3_ at pH 12.0.


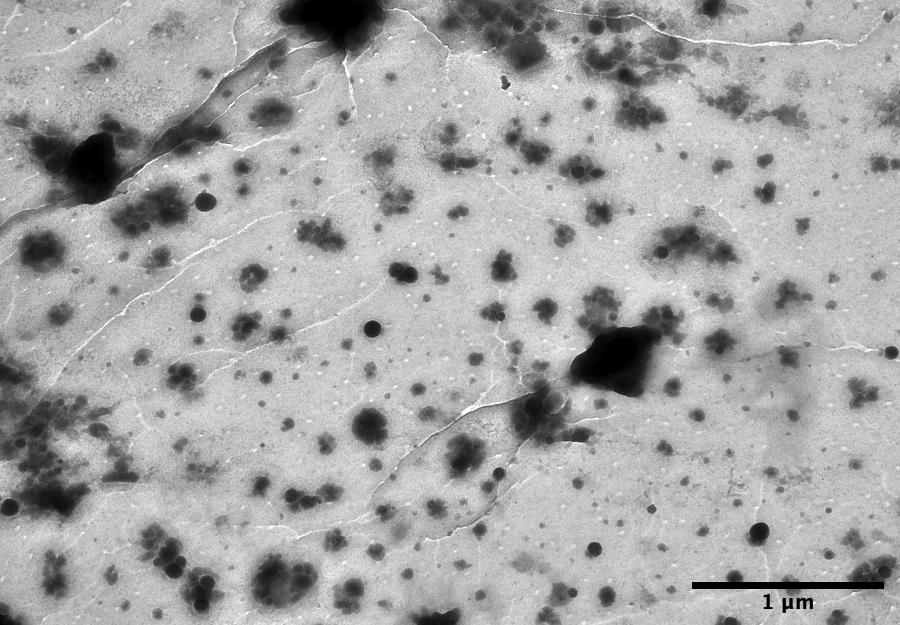
Figure 13. Confocal micrograph of DA/GOH in 50 mM NaHCO_3_ at pH 11.9.
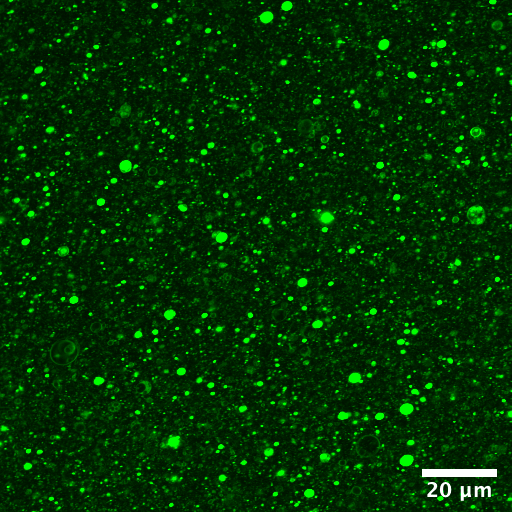
Figure 14. Confocal micrograph of DA/DOH in H_2_O under anoxic conditions at pH 12.1.
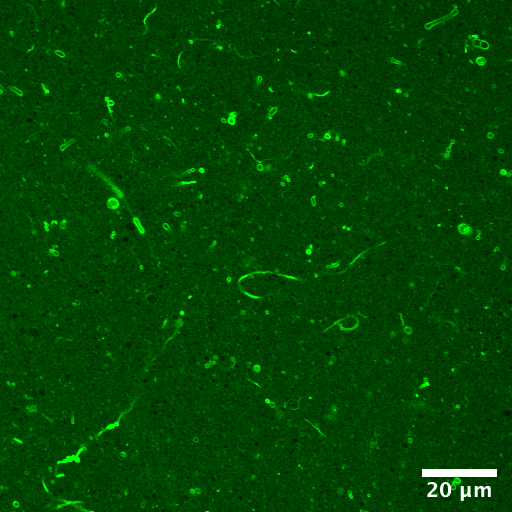
Figure 15. Confocal micrograph of DA/GOH in H_2_O under anoxic conditions at pH 12.1.
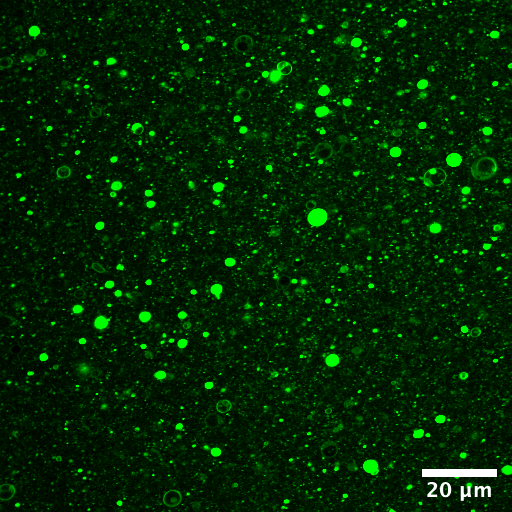
Figure 16. Confocal micrograph of DA/DOH in 1 mM FeCl_2_ under anoxic conditions at pH 12.1.
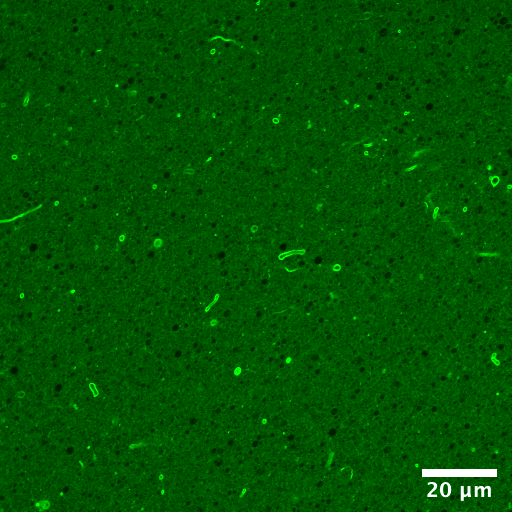
Figure 17. Confocal micrograph of DA/GOH in 1 mM FeCl_2_ under anoxic conditions at pH 12.1.
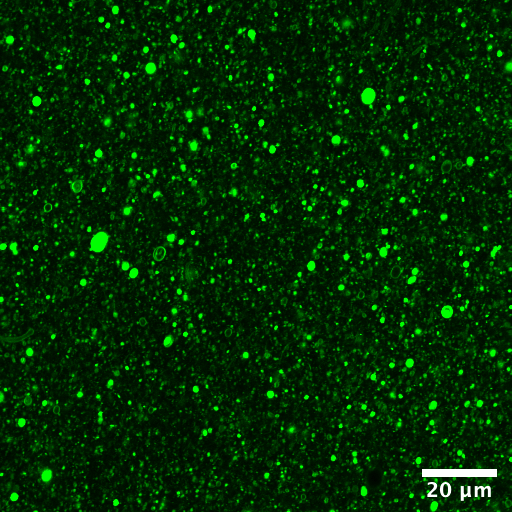
Figure 18. Confocal micrograph of DA/DOH in 1 mM Na_2_S under anoxic conditions at pH 12.1.
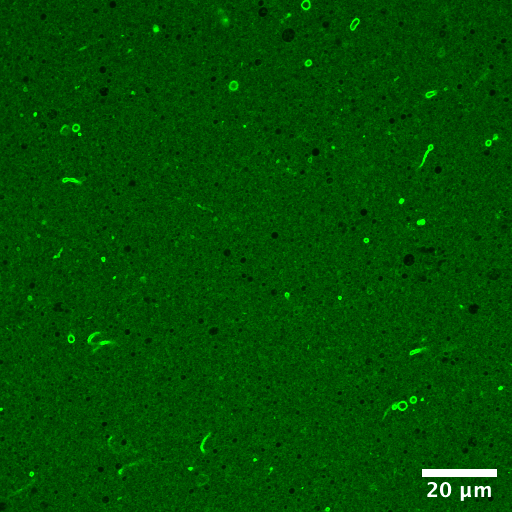
Figure 19. Confocal micrograph of DA/GOH in 1 mM Na_2_S under anoxic conditions at pH 12.0.
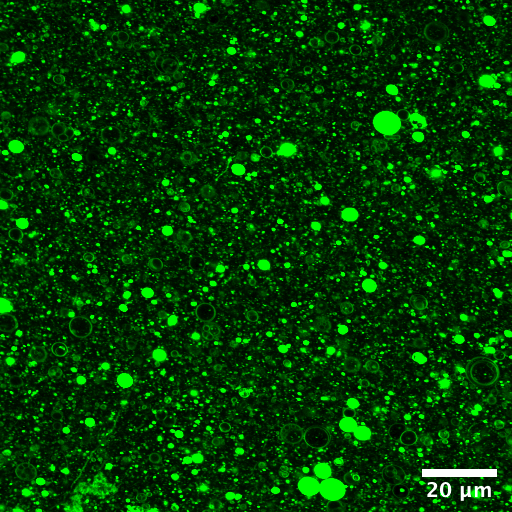
Figure 20. Confocal micrograph of DA/DOH in 0.5 mM Fe(II)S_2_ under anoxic conditions at pH 12.1.


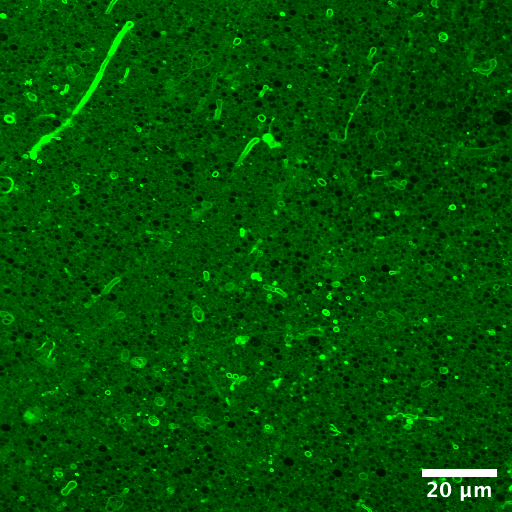


Figure 21. Confocal micrograph of DA/GOH in 0.5 mM Fe(II)S_2_ under anoxic conditions at pH 12.0.
